# *Oligella ureolytica* Bacteremia in Elderly Woman, United States

**DOI:** 10.3201/eid2107.150242

**Published:** 2015-07

**Authors:** Tristan Simmons, Eryn Fennelly, David Loughran

**Affiliations:** Philadelphia College of Osteopathic Medicine, Philadelphia, Pennsylvania, USA

**Keywords:** *O. ureolytica*, *Oligella ureolytica*, CDC Group IVe, bacteremia, United States, bacteria

**To the Editor:** *Oligella ureolytica* is an aerobic gram-negative coccobacillus found as a commensal organism in human urinary tracts (*1*). Previously referred to as CDC Group IVe, this bacterium is not commonly encountered as a source of infection and is difficult to isolate by using conventional laboratory procedures (*2*). The few cases of pathogenic infection with *O. ureolytica* described in the literature have occurred in patients ranging in age from newborn to 89 years and from the varied locations of India, Turkey, Canada, and the United States (*3**–**7*). We report a case of *O. ureolytica* bacteremia in a patient in whom sepsis was diagnosed and review the current literature on this emerging pathogen.

A 66-year-old woman sought treatment in our emergency department for a fever of 100.7°F, femur fracture, and a right buttock stage III decubitus ulcer. She reported having fallen 4 days earlier, after which she was unable to walk and spent 4 days laying in her own urine and feces. Blood tests revealed an elevated leukocyte count of 24.4 × 10^9^ cells/L (76% neutrophils, 2% bands), and urinalysis showed trace leukocyte esterase, +3 bacteria, and 5–10 leukocytes. Chest radiograph and head computed tomography images were unremarkable. Her electrocardiogram showed nonspecific ST wave changes. Samples from the patient’s blood, urine, and wounds were collected while the patient was in the emergency department and were sent for culture.

Wound cultures showed growth of *Proteus mirabilis* and *Enterococcus* spp. The urine culture grew >100,000 CFU *Escherichia coli*. The first set of blood cultures grew *O. ureolytica* in aerobic and anaerobic bottles, but another set drawn 30 min later showed no growth. The blood cultures were processed by using the Bact/Alert 3D (bioMérieux, Marcy l’Etoile, France) and Gram stained. Identification was from the Vitek 2 compact system (bioMérieux). The *O. ureolytica* sample was sensitive to amikacin, ampicillin/sulbactam, ceftazidime, ceftriaxone, gentamicin, imipenem, levofloxacin, nitrofurantoin, trimethoprim/sulfamethoxazole, and chloramphenicol. No resistance was found.

Because of the unique bacteremia, further diagnostics were conducted. The results of chest, abdomen, and pelvic computed tomography scans were unremarkable. HIV test results were negative. The nonspecific electrocardiogram changes prompted us to request a transesophageal echocardiogram, but the patient refused. For 10 days, the patient was given vancomycin (1 g/d), aztreonam (2 g/8 h), and metronidazole (500 mg/8 h). Cultures of blood that had been collected 5 and 8 days after the original culture were sterile. After 16 days, leukocytosis and fever had resolved, and the patient was discharged to a skilled nursing facility. Although we found no reports in the literature of endocarditis caused by *O. ureolytica*, the patient’s refusal of a transesophageal echocardiogram and the presence of the uncommon bacterium led us to empirically continue aztreonam for endocarditis after her discharge.

The literature reports 5 cases of pathogenic *O. ureolytica* infection (Table). This bacterium has also been isolated from the respiratory tract of patients with cystic fibrosis (*9*). A 2-year study conducted in 1983 at a high-volume hospital in the United States demonstrated *O. ureolytica* growth in the urine of 72 patients (*8*). Of these patients, 71 had long-term urinary drainage systems and 14 had symptomatic urinary tract infections. Many of these patients were permanently disabled from spinal cord injuries (*8*). This study was the only one we found focused on *O. ureolytica* infection in the clinical setting. We found no cases in which a patient’s death was attributed to *O. ureolytica* infection, and all reported cases resolved with antimicrobial drug treatment (*3**–**8*). The low virulence of this organism may contribute to the paucity of recognized cases.

**Table:** Documented cases of pathogenic *Oligella ureolytica* infection*

Year	Patient age, y	Patient sex	Location	Culture source	Concurrent conditions	Urinary disorder	Reference†
2014	30	M	India	Blood	Metastatic lung adenocarcinoma	Urinary incontinence	(*3*)
2013	Newborn	F	Turkey	Blood	None	Maternal urine exposure during delivery?	(*4*)
2013	89	M	United States	Urine	Adenocarcinoma of prostate	High post void residual	(*5*)
1996	49	F	Canada	Neck lymph node	Non-Hodgkin lymphoma	None	(*6*)
1993	40	M	United States	Blood	AIDS, sacral ulcer, diarrhea	None	(*7*)

*Some published cases that were believed to be contamination or for which the organisms did not fit the laboratory profile of *O. ureolytica* were excluded. †Antimicrobial drug sensitivity has varied among reports; some resistant organisms have been encountered (*3**–**8*).

Of the reported cases, all occurred as opportunistic infections in patients with a source of immunosuppression such as malignancy, HIV, or newborn status. The patient we reported in this article showed no evidence of malignancy and had no major source of immunosuppression besides malnutrition, tobacco use, and advanced age. The patient’s wound had been contaminated by urine and feces, which was postulated to be the cause of bacteremia in the 1993 case.

Limitations in commonly available laboratory procedures make the identification of this bacterium difficult. The incubation period is long (4 days), and not all laboratories incubate cultures for that long, as occurred in the 2013 urinary tract infection case (*1**,**3**,**5*). Also, the identification of less commonly encountered bacteria is not always pursued to the genus and species level (*2*). Furthermore, it is believed that *Oligella* spp. can be misidentified as phenotypically similar organisms, such as *Bordetella bronchiseptica* and *Achromobacter* spp. (*4*,*10*).

We believe that many cases of *O. ureolytica* infection have gone unrecognized or were incorrectly identified. Some cases may also have been dismissed as contamination because of laboratorians’ and clinicians’ lack of familiarity with this bacterium. Our review suggests that advancing laboratory techniques will lead to more recognized cases and that further studies are necessary to understand this bacterium’s clinical significance.
